# A systematic review and meta-analysis of sacubitril-valsartan in the treatment of ventricular remodeling in patients with heart failure after acute myocardial infarction

**DOI:** 10.3389/fcvm.2022.953948

**Published:** 2022-10-11

**Authors:** Xiaomin Zhou, Hongjun Zhu, Yawei Zheng, Xiaodong Tan, Xinyu Tong

**Affiliations:** ^1^Nanjing University of Chinese Medicine, Nanjing, China; ^2^Department of Cardiology, Wuxi Hospital Affiliated Nanjing University of Chinese Medicine, Wuxi, China

**Keywords:** sacubitril-valsartan, ventricular remodeling, heart failure, acute myocardial infarction, meta-analysis, systematic review

## Abstract

**Objective:**

To systematically review the efficacy and safety of sacubitril and valsartan in treating acute myocardial infarction complicated with heart failure and to observe whether it can further improve patients’ cardiac function, delay left ventricular remodeling, and reduce major adverse cardiovascular events (MACEs).

**Methods:**

Electronic databases including Pubmed, Embase, the Web of Science, Cochrane Library, Scopus, CNKI, Wanfang Data, and VIP were searched. The search period was from the establishment of the database to March 2022 to search for relevant controlled trials. Two investigators independently screened the literature, extracted data, and assessed the risk of bias. Revman5.3 and Stata14 software were used for statistical analysis.

**Results:**

A total of 13 studies, with 6,968 patients were included. Meta-analysis results showed that sacubitril-valsartan increased left ventricular ejection fraction (LVEF) and decreased NT-proBNP level was better at 6 months and within 3 months of follow-up compared with the control group (*P* < 0.00001), but there was no significant difference at the 12-month follow-up (*P* > 0.05). Sacubitril-valsartan reducing LVEDD [*MD* = −2.55, 95%CI(−3.21, −1.88), *P* < 0.00001], LVEDVI [*MD* = −3.61, 95%CI(−6.82, −0.39), *P* = 0.03], LVESVI [*MD* = −3.77, 95%CI(−6.05, −1.49), *P* = 0.001], and increasing the distance of the 6-min walk test [*MD* = 48.20, 95%CI(40.31, 56.09), *P* < 0.00001] were more effective. Compared with ACEI/ARB, the use of ARNI can further reduce the total incidence of adverse cardiovascular events [RR = 0.72, 95%CI(0.62, 0.84), *P*<0.0001] and the rate of HF rehospitalization [RR = 0.73, 95%CI(0.61, 0.86), *P* = 0.0002] in patients with acute myocardial infarction and heart failure; there was no significant difference in the incidence of cardiac death, recurrence of myocardial infarction, and malignant arrhythmia between the experimental group and the control group (*P* > 0.05). In terms of the incidence of adverse reactions, the incidence of cough in ARNI was lower than that in ACEI/ARB group [RR = 0.69, 95%CI(0.60, 0.80), *P* < 0.00001], but the incidence of hypotension was higher [RR = 1.29, 95%CI(1.18, 1.41), *P* < 0.00001], and the adverse reactions of hyperkalemia, angioedema and renal insufficiency were not increased (*P* > 0.05).

**Conclusion:**

The use of sacubitril-valsartan sodium in patients with acute myocardial infarction complicated with heart failure can significantly improve cardiac function and reverse ventricular remodeling, reducing the risk of re-hospitalization for heart failure. There is no apparent adverse reaction except easy cause hypotension.

**Systematic trial registration:**

[www.ClinicalTrials.gov], identifier [CRD42022322901].

## Introduction

Primary myocardial damage is the leading cause of heart failure (HF) (1). According to epidemiological studies (2), the incidence of signs and symptoms of HF after acute myocardial infarction (AMI) is about 25%, and about 40% of myocardial infarction (MI) is accompanied by left ventricular systolic dysfunction. Data shows that the incidence of HF in the short and long-term follow-up of AMI patients in Minnesota is 24–41% (3). The Framingham Heart Study showed that the 30-day incidence of HF after MI increased from 10 to 23.1% within two decades, and the 5-year incidence increased from 27.6 to 31.8% (4). Among several hospitals in Argentina, the incidence of HF in ST-elevation myocardial infarction (STEMI) patients is the most common, accounting for 43.0% (5). There is a relatively limited amount of epidemiological data on post-MI HF in China. A Chinese multi-center large-sample study showed that the incidence of HF 7 days after MI in STEMI patients (62.4% received reperfusion therapy) was 19.3% (6). The BRIGHT study found that the incidence of HF on admission was 14.3% in 2,194 patients with AMI who underwent emergency percutaneous coronary intervention (PCI) (7). Therefore, HF is a common complication of MI with or without reperfusion therapy.

Although the level of medical care for MI has improved, its death rate has been decreasing yearly. Nevertheless, the development of HF during admission is a severe complication. The occurrence and progression of HF after MI triples the risk of overall mortality and quadruple cardiovascular mortality (8). The combination of these two diseases also impacts adverse events, as it significantly increases the risk of reinfarction, stroke, ventricular arrhythmia, cardiogenic shock, and death (9, 10). In short, reducing infarct size and improving overall ventricular function after myocardial reperfusion has important prognostic implications.

In the long term, the best treatment for heart failure after MI is neurohormonal blockade (11). Sacubitril-valsartan is an angiotensin receptor-neprilysin inhibition, which works by inhibiting angiotensin receptor neprilysin and enkephalinase from modulating the neurohormonal axis. It improves neurohormonal balance more than blocking the renin-angiotensin-aldosterone system (RAAS) alone (12). In the sizeable PARADIGM-HF trial, sacubitril-valsartan reduced the number of hospitalizations for heart failure and decreased mortality in patients with chronic HFrEF compared with conventional ACEI/ARB (13). The latest RCT (PARADISE-MI) showed that sacubitril-valsartan compared with ramipril did not significantly reduce the incidence of heart failure or cardiovascular death after myocardial infarction, but it still has further improvements (14). There are now a sufficient number of controlled trials demonstrating the efficacy of sacubitril-valsartan in the treatment of myocardial infarction with heart failure. However, no studies have systematically reviewed this literature to provide comprehensive insights. Therefore, we performed a systematic review and meta-analysis to compare the clinical outcomes of sacubitril-valsartan in patients with heart failure after acute myocardial infarction.

## Methods

The study protocol was registered with PROSPERO (CRD42022322901) and performed according to the PRISMA (Preferred Reporting Items for Systematic reviews and Meta-analyses) guidelines (15).

### Search strategy

Literatures were searched in PubMed, Embase, Cochrane Library, Web of Science, Scopus, and China National Knowledge Infrastructure (CNKI), Wanfang Data knowledge service platform (WanFang Data), and VIP information resource integration service platform (VIP) without any restrictions from inception to March 2022. The search strategy included the following keywords: angiotensin receptor-neprilysin inhibition, sacubitril valsartan, LCZ696, Entresto, ARNI, Heart Failure, Myocardial Infarction, and ST Elevation Myocardial Infarction.etc (Supplementary Table 1). Moreover, we manually checked the reference list of retrieved articles to identify the potentially relevant studies.

### Study selection

Studies were included if they met the following criteria: (i) Hemodynamically stable patients with HF after AMI: (ii) The type of study design included randomized controlled trial (RCT) and non-randomized controlled trial (non-RCT): (iii) Adult (age > 18 years) patients were treated with conventional treatment, the control group remained unchanged based on conventional treatment or given angiotensin-converting enzyme inhibitor (ACEI)/angiotensin receptor blocker (ARB) treatment, the experimental group was given sacubitril/valsartan tablets based on conventional treatment; (iv) Studies reported the primary or secondary outcomes.

The exclusion criteria were as follows: (a) observation, cohort, case-control, case-series, qualitative studies, uncontrolled trials, and laboratory studies; (b) studies were duplicated publications; (c) studies without useable data; (d) pediatric, animal, or cell studies.

### Outcomes

Primary outcomes: Left myocardial remodeling parameters (LVEF, LVESVI, LVEDVI, LVEDD) and Major Adverse Cardiovascular Events (MACEs). *MACEs* comprise cardiac death, hospitalization for recurrent heart failure, recurrent myocardial infarction, and malignant arrhythmia.

Secondary outcomes: N-terminal pro-brain natriuretic Peptide (NT-proBNP) and 6-min walking distance (6MWD).

The safety outcomes include adverse events (hypotension, neurologic edema, hyperkalemia, dry cough, liver, and kidney dysfunction).

### Study screening and data extraction

Two investigators independently screened literature, extracted data, and cross-checked it according to pre-determined inclusion and exclusion criteria. In case of disagreements, they were resolved through discussion or with the assistance of a third investigator. Firstly, using literature management software to exclude duplicate literature, the titles and abstracts of literature were read for preliminary screening to exclude irrelevant literature; If necessary, contact the original study authors by phone or email for undetermined but essential information. We were using a pre-established data extraction table to extract the data of the included studies, including the article title, the name of the first author, the year of publication, the basic information of the subjects, intervention measures and courses of treatment, and outcome indicators.

### Risk of bias assessment

Two investigators independently assessed the risk of bias in the included studies, and the results were cross-checked. The risk of bias was assessed using the RCT risk of bias assessment tool recommended in Cochrane Handbook 5.1.0 (16, 17). The evaluation content includes seven aspects: randomization method, allocation concealment, blinded implementation of subjects and intervention providers, blinded implementation of outcome evaluation, completeness of outcome data, selective reporting, and other sources of bias. According to the influence of material biases, the risk of bias was then adjudicated with low, high, or unclear levels.

### Statistical analysis

Statistical analysis was performed using RevMan5.3 and Stata14.0 software. MD (mean difference) was used as effect analysis statistic for measurement data, and SMD (standard mean difference) was used if different measurement methods or units were used; RR (risk ratio) was used as effect analysis statistic for dichotomous variables of effect measurement indicators, and each effect size provided its 95% CI. The heterogeneity among the results of the included studies was analyzed by the χ^2^ test (α = 0.1), and the size of the heterogeneity was quantitatively judged by *I*^2^. Suppose the heterogeneity among the results of each study was small (*P* ≥ 0.1 and *I*^2^ ≤ 50%), a fixed-effects model was used; otherwise (*P*<0.1 or *I*^2^>50%), a random-effects model was used for meta-analysis. Significant clinical heterogeneity was handled using methods such as subgroup analysis, sensitivity analysis, or only descriptive analysis. When the number of studies was ≥ 10, funnel plots were drawn to assess the possibility of publication bias. Funnel plots with asymmetric distributions indicate a high potential for publication bias. In addition, Egger’s test analyzed the possibility of publication bias, and *P* > 0.1 indicated that the possibility of publication bias was slight. Finally, to assess the stability of the results, we performed a sensitivity analysis by excluding included studies one by one.

## Results

### Search results and study characteristics

**FIGURE 1:**
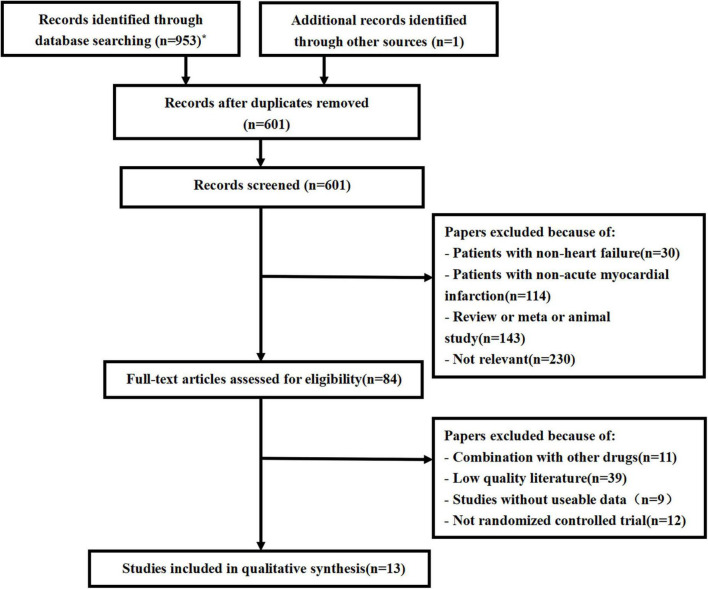
Flow chart for literature screening (PRISMA Flow Diagram). *PubMed (79); Embase (137); Web of science (159); Cochrane library (185); Scopus (176); CNKI (91); WanFang (80); VIP (46).

We conducted the last search on March 7, 2022, and the results of our literature search are shown in Figure 1. A preliminary search yielded 953 relevant sources, and after the layer-by-layer screening, 13 studies (14, 18–29) were ultimately included. A total of 6,968 patients with AMI and HF were included in the final group of included literature, including 3,483 in the experimental group and 3,485 in the control group. In each study, there were no significant differences between the treatment and control groups regarding age and gender. Eleven of these studies used ACEI/ARB in the control group and sacubitril/valsartan in the experimental group. In the other two studies, the control group did not use ACEI/ARB, and the experimental group was supplemented with sacubitril and valsartan based on the control group. In addition, follow-up mostly lasted for 3, 6, and 12 months. The characteristics of the included studies are shown in Table 1.

**TABLE 1:** The characteristics of the included studies.

Study	Sample size		Age (years, mean ± *SD*)		Male/Female		Drugs		Follow-up (months)	Design	Outcomes
	ARNI	Control	ARNI	Control	ARNI	Control	ARNI	Control			
Zornoff Leonardo et al. (11)	84	86	62.29 ± 12.82	63.49 ± 11.61	52/32	56/30	Sacubitril/valsartan, MTD	Valsartan 80 mg, qd	12	non-RCT	➀➄➆➇
Volpe et al. (12)	42	39	51.30 ± 6.21	51.28 ± 6.27	24/15	27/15	Sacubitril/valsartan 200 mg, bid + Control	Bisoprolol 1.25 mg, qd	6	non-RCT	➀➃➄➅➇
McMurray et al. (13)	50	50	53.12 ± 9.08	55.5 ± 12.50	35/15	38/12	Sacubitril/valsartan 100 mg, bid	Ramipril 5 mg, bid	6	RCT	➀➄➆
Jering et al. (14)	47	46	61.80 ± 10.60	59.7 ± 10.10	42/5	43/3	Sacubitril/valsartan 200 mg, bid	Valsartan 160 mg, bid	12	RCT	➀➁➂➃
Han et al. (7)	2,830	2,831	64.0 ± 11.60	63.5 ± 11.40	2,167/ 663	2,131/ 700	Sacubitril/valsartan 200 mg, bid	Ramipril 5 mg, bid	43	RCT	➆➇
Liberati et al. (15)	68	69	59.13 ± 7.15	60.56 ± 7.62	52/16	54/15	Sacubitril/valsartan, MTD	Enalapril,MTD	6	RCT	➀➁➂➃➆➇
Zeng et al. (16)	45	45	54.09 ± 8.26	53.34 ± 7.65	39/6	37/8	Sacubitril/valsartan 50 mg, bid	Perindopril 8 mg, qd	3/6/12	RCT	➀➃➄
Higgins and Green (17)	80	80	59.00 ± 10.30	58.00 ± 10.40	69/11	67/13	Sacubitril/valsartan, MTD	Valsartan 40 mg, qd	3/6	RCT	➀➁➂➃➆➇
Ye et al. (18)	48	50	60.00 ± 6.00	60.00 ± 5.00	35/13	38/12	Sacubitril/valsartan 200 mg, bid	Enalapril 10 mg, bid	6	RCT	➀➃➄➅➆
Chen et al. (19)	30	30	55.40 ± 10.10	54.60 ± 10.30	15/15	15/15	Sacubitril/valsartan, MTD	Enalapril,MTD	1/3/6	RCT	➀➃➄
Rezq et al. (20)	76	76	62.40 ± 4.30	63.10 ± 4.20	42/34	44/32	Sacubitril/valsartan, MTD + Control	Conventional treatment	2	RCT	➀➄➅➇
Docherty et al. (21)	43	43	48.60 ± 10.40	49.80 ± 7.20	35/8	37/6	Sacubitril/valsartan 200 mg, bid	Benazepril 10 mg, qd	3	non-RCT	➀➁➂➃➅➆➇
Wang and Fu (22)	40	40	63.90 ± 8.20	62.00 ± 7.60	35/13	38/12	Sacubitril/valsartan 200 mg, bid	Valsartan 80 mg, qd	3/6	RCT	➀➃➄➆

ARNI, angiotensin receptor-neprilysin inhibitor; MTD, maximum tolerated; *SD*, standard deviation; Outcomes: ➀LVEF; ➁LVESVI; ➂LVEDVI; ➃NT-proBNP; ➄LVEDD; ➅6MWT; ➆MACEs; ➇Adverse reactions.

### Risk of bias in the included studies

**FIGURE 2:**
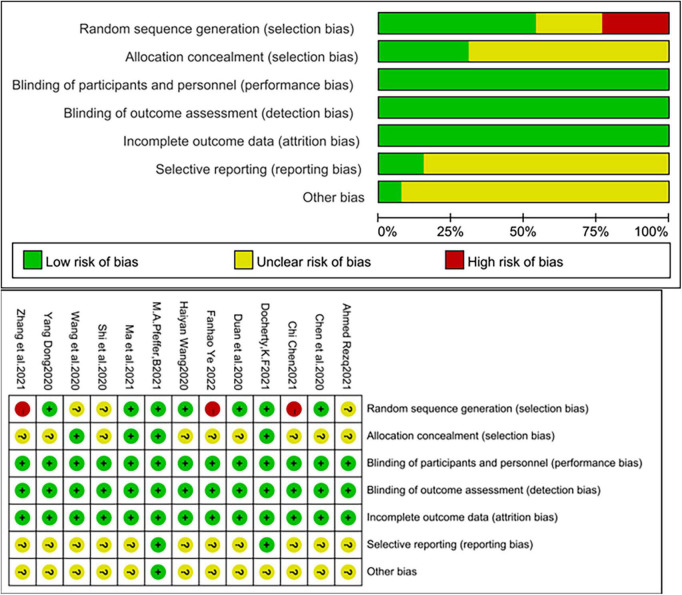
Risk of bias in the included studies.

The quality of the included studies was relatively high. Of these, three studies were assigned at the discretion of the clinician (18, 19, 28), two studies used an interactive web for grouping and assignment concealment (14, 21), and two studies used sealed envelopes for randomization and assignment concealment (15, 20), three studies used random number tables for randomization (25, 26, 29). The remaining studies mentioned randomization but did not mention the specific method and did not mention allocation concealment in the text. Three studies were multicenter, double-blind trials (14, 17, 21). Although patients, experimenters, and outcome assessors were not blinded in the remaining trials, the judgment of outcome indicators would not be affected by the unblinded method. In summary, the risk of bias in the included studies was relatively low (Figure 2).

### Left myocardial remodeling parameters

#### Left ventricular ejection fraction

**FIGURE 3:**
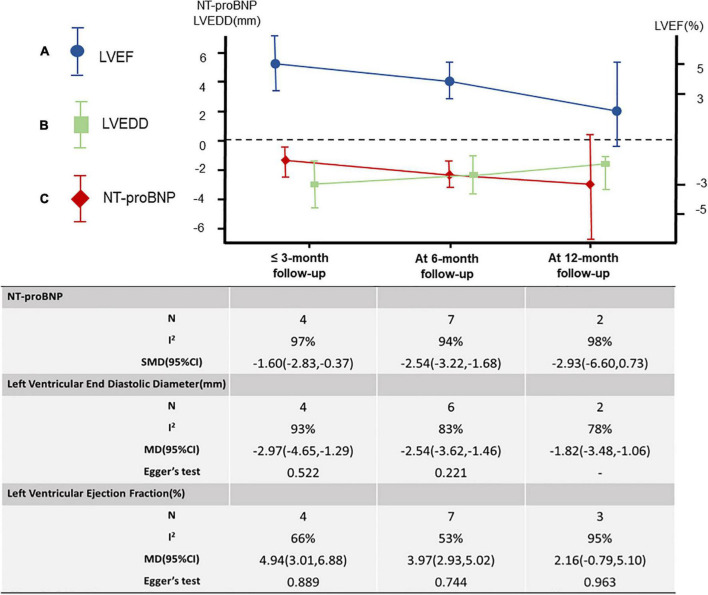
**(A–C)** Meta-analysis of LVEF, LVEDD, and NT-proBNP under different treatment courses.

A total of 12 studies (18–27) were included, including 2,868 patients. Meta-analysis results of the random effects model show that sacubitril-valsartan sodium tablets can improve the level of left ventricular ejection fraction (LVEF) [*MD* = 3.87, 95%CI(2.80, 4.94, *P*<0.00001]. Subgroup analysis was carried out with different courses of treatment, and the results showed that: the course of treatment was effective at 6 months [*MD* = 3.97, 95%CI(2.93, 5.02), *P* < 0.00001] and within 3 months [*MD* = 4.94, 95%CI(3.01, 6.88), *P* < 0.00001], while the course of treatment for 12 months (*P* = 0.15) had no significant difference compared with the control group (Figure 3A).

#### Left ventricular end-diastolic diameter

Seven studies were included (18, 20, 23, 25–27, 29), including 831 patients. The results of the random effects model meta-analysis showed that sacubitril-valsartan sodium tablets were better in reducing left ventricular end-diastolic diameter (LVEDD) [*MD* = −2.55, 95%CI(−3.21, −1.88), *P*<0.00001]. Subgroup analysis was conducted with different courses of treatment, and the results showed that: the course of treatment was effective at 12 months [*MD* = −1.82, 95%CI(−3.48, −0.16), *P* = 0.03], 6 months [*MD* = −2.54, 95%CI(−3.62, −1.46), *P*<0.00001] and 3 months [*MD* = −2.97, 95%CI(−4.65, −1.29), *P* = 0.0005], and the difference were statistically significant (Figure 3B).

#### Left ventricular end-systolic volume index

**FIGURE 4:**
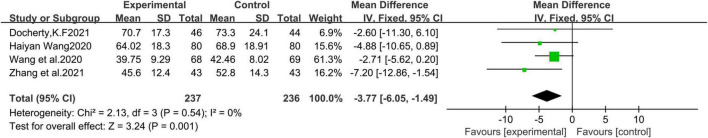
Meta-analysis for left ventricular end-systolic volume index.

A total of 4 studies (21, 22, 24, 28) with 473 patients were included. Meta-analysis results of the fixed effects model show that sacubitril-valsartan sodium tablets can reduce left ventricular end-systolic volume index (LVESVI) [*MD* = −3.77, 95%CI(−6.05, −1.49), *P* = 0.001] (Figure 4).

#### Left ventricular end-diastolic volume index

**FIGURE 5:**
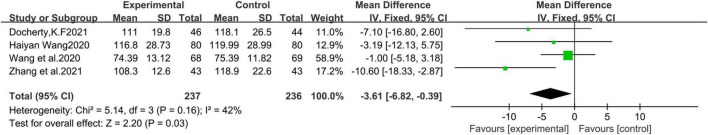
Meta-analysis for left ventricular end-diastolic volume index.

A total of 4 studies (21, 22, 24, 28) were included, including 473 patients. Fixed effects model Meta-analysis results show that sacubitril and valsartan sodium tablets can reduce left ventricular end-diastolic volume index (LVEDVI) [MD = −3.61, 95%CI(−6.82, −0.39), *P* = 0.03] (Figure 5).

### Major adverse cardiovascular events

**FIGURE 6:**
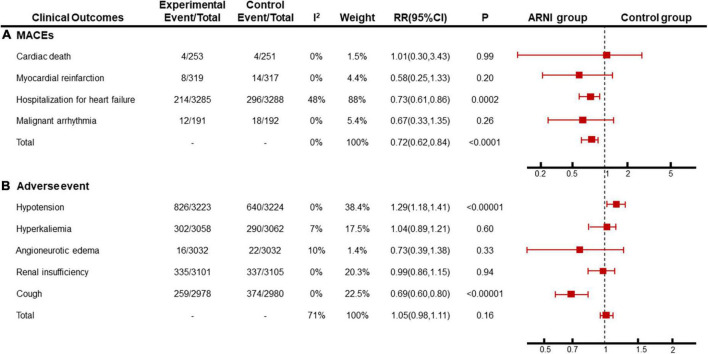
Meta-analysis results of **(A)** MACEs and **(B)** adverse event. Subgroup analysis was performed with different outcomes.

We summarized cardiac death, recurrent myocardial infarction, hospitalization for recurrent heart failure, and malignant arrhythmia as adverse cardiovascular events. Among them, 5 articles (14, 19, 20, 22, 28) describe the incidence of cardiac death, and 6 articles (19, 20, 22, 27–29) describe the incidence of recurrent myocardial infarction, 9 articles (14, 18–20, 22, 24, 25, 28, 29) describe the incidence of hospitalization for recurrent heart failure, 3 articles (22, 24, 28) describe the incidence of malignant arrhythmia describe. The heterogeneity among the studies was small, and a fixed-effects model was used for Meta. The results showed that there was no significant difference in the incidence of cardiac death [RR = 1.01, 95%CI(0.30, 3.43), *P* = 0.99], recurrence of myocardial infarction [RR = 0.58, 95%CI(0.25, 1.33), *P* = 0.20], and malignant arrhythmia [RR = 0.67, 95%CI(0.33, 1.35), *P* = 0.26] between the experimental group and the control group. The hospitalization rate of recurrent heart failure [RR = 0.73, 95%CI(0.61, 0.86), *P* = 0.0002] and the total incidence of adverse cardiovascular events [RR = 0.72, 95%CI(0.62, 0.84), *P*<0.0001] in the experimental group were lower than those in the control group, and the difference was statistically significant (Figure 6A).

### N-terminal pro-brain natriuretic peptide

A total of 9 studies (19, 21–26, 28, 29) were included, including 874 patients. Because the units used in each literature were different, we used the standardized mean difference (SMD) to measure the results. The results of the random effects model meta-analysis showed that sacubitril-valsartan sodium tablets were more effective in reducing the level of NT-proBNP [SMD = −2.26, 95%CI(−2.91, −1.60), *P*<0.00001]. Subgroup analysis was carried out with different courses of treatment, and the results showed that: the course of treatment was effective at 6 months [SMD = −2.45, 95%CI(−3.22, −1.68), *P*<0.00001] and within 3 months [SMD = −1.60, 95%CI(−2.83, −0.37), *P* = 0.01], while the course of treatment for 12 months (*P* = 0.12) had no significant difference compared with the control group (Figure 3C).

### Six-min walk distance

**FIGURE 7:**
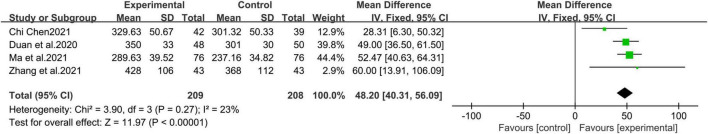
Meta-analysis for six-min walk distance.

A total of 4 studies (19, 25, 27, 28) with 417 patients were included. Fixed effects model meta-analysis results show that sacubitril and valsartan sodium tablets can increase walking distance in 6 min [*MD* = 48.20, 95%CI(40.31, 56.09), *P*<0.00001] (Figure 7).

### Adverse events

Clinically, the adverse reactions with a higher incidence of ARNI and ACEI/ARB include hypotension, hyperkalemia, angioedema, cough, and liver and kidney dysfunction. in this study, 7 papers (14, 18, 19, 22, 24, 27, 28) described the incidence of hypotension (*P* < 0.00001, *I*^2^ = 0%), 4 papers (14, 18, 22, 27) described the incidence of hyperkalemia (*P* = 0.60, *I*^2^ = 7%), 4 articles (14, 18, 19, 27) describe the incidence of angioedema (*P* = 0.33, *I*^2^ = 10%), 5 articles (14, 18, 22, 27, 28) describe the incidence of renal insufficiency (*P* = 0.94, *I*^2^ = 0%), and 3 articles (14, 22, 24) described the incidence of cough (*P* < 0.00001, *I*^2^ = 0%), and the incidence of overall adverse reactions was not statistically different (*P* = 0.16, *I*^2^ = 71%). Subgroup analysis showed that the heterogeneity of the total adverse event rate was derived from different clinical symptoms, and the homogeneity among subgroups was good. A fixed effect model was used for meta-analysis. the results showed that the incidence of cough in the sacubitril-valsartan group was lower than that in the ACEI/ARB group [RR = 0.69, 95%CI(0.60, 0.80)], but the incidence of hypotension in the control group was lower than that in the ARNI group [RR = 1.29, 95%CI(1.18, 1.41)], and there was no significant difference in other adverse reactions between the two groups (Figure 6B).

### Sensitivity analysis

Sensitivity analysis was carried out through seriatim excluding one trial each time and re-performing meta-analysis of the remaining trials. After excluding Zhang’s article, there was no significant difference in LVEDVI between the experimental group and the control group (*P* = 0.23). After excluding Haiyan Wang’s article, there was no significant difference in NT-proBNP between the experimental group and the control group within 3 months of follow-up (*P* = 0.08). These changes are thought to be caused by a reduction in sample size. After excluding the articles of Docherty K.F, the difference between LVEF (*P* = 0.02) and NT-proBNP (*P* < 0.00001) between the experimental group and the control group became significant at the 12-month follow-up. However, this difference lacks clinical value. There were no significant changes in other results and statistical heterogeneity after item by item exclusion, indicating that the results were stable.

### Publication bias

**FIGURE 8:**
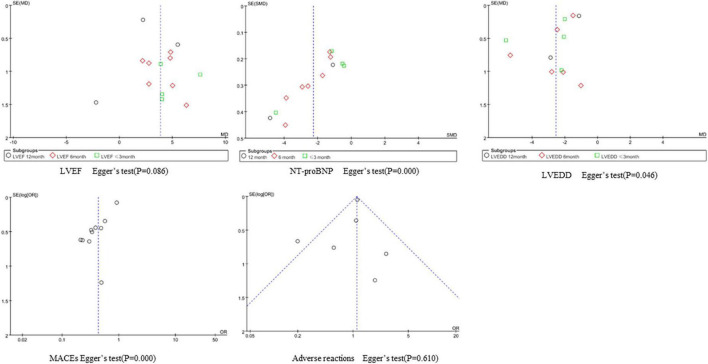
Funnel plot of publication bias.

We performed a funnel plot and Egger’s test to observe publication bias for indicators with a large number of included studies, and the results are as follows. The incidence of adverse reactions is less likely to be subject to publication bias. The funnel plots of LVEF and LVEDD were symmetrical, but Egger’s test *P* < 0.1, suggesting potential publication bias. The funnel plots of NT-proBNP and MACEs showed strong asymmetry, suggesting the possibility of publication bias, which may be related to the greater direct sample differences (Figure 8).

## Discussion

Despite major advances in the treatment of both coronary artery disease and HF, AMI remains the leading cause of HF. Cardiomyocytes undergo structural changes leading to progressive death within 30 min of ischemia, and the resulting inflammatory response also contributes to the development of HF (30). Although PCI can minimize acute myocardial ischemia/reperfusion injury (IRI) and is the most effective way to perform myocardial reperfusion (31), reperfusion itself can cause a second wave of injury by producing reactive oxygen species, leading to myocardial necrosis, reactive myocardial hyperplasia, myocardial fibrosis, and other pathological processes resulting in HF and ventricular remodeling (32). After STEMI is combined with HF, the systolic function of the heart decreases, and coronary perfusion decreases. This not only aggravates myocardial ischemia (MI), but also reduces the effective blood circulation of the whole body, resulting in excessive activation of neuroendocrine pathways (33). Therefore, new therapies are needed to alleviate the clinical outcomes of HF after AMI.

Sacubitril-valsartan (LCZ696) is an angiotensin receptor enkephalinase inhibitor (ARNI) class of drugs, which acts by blocking angiotensin II receptors and inhibiting enkephalinase (NEP). It can dilate blood vessels, prevent and reverse cardiovascular remodeling and promote natriuresis (34). Sacubitril-valsartan has been shown to improve cardiac insufficiency (35), hypertension (36), ventricular arrhythmia (37), and chronic kidney disease (38) in experimental and clinical studies. In the setting of AMI, the onset of acute myocardial ischemia induces a proinflammatory response that is exacerbated by myocardial reperfusion after PPCI. According to the mouse experiments of Masanobu (39), it was predicted that LCZ696 could inhibit pro-inflammatory cytokines, matrix metalloproteinase-9 activity, and aldosterone production, and clinically help to improve the survival rate after acute myocardial infarction.

Gender is a risk factor for cardiovascular disease. Therefore, it is meaningful to compare the efficacy of sacubitril-valsartan in patients of different genders. In subgroup analyses of the PARAGON-HF trial, we found that only sex and LVEF contributed to the differences in results (40). Sacubitril-valsartan has a better therapeutic effect on low LVEF value and women. A study published in Circulation showed that sacubitril-valsartan was significantly more beneficial in women than valsartan (41). Specifically, it reduced the hospitalization rate for recurrent heart failure. Sun and Tao demonstrated that sacubitril-valsartan could effectively reverse ventricular remodeling in patients with acute anterior myocardial infarction, and the treatment effect was better in women than in men (42, 43). The reason may be that if women are viewed as having deficient cGMP–protein kinase G signaling, sacubitril and valsartan can increase their natriuretic peptide levels.

Ventricular remodeling refers to changes in the ventricle structure. As adverse remodeling progresses, echocardiography shows atrial and ventricular lengthening, increases in volume and mass, as well as deterioration in systolic and diastolic function (44). The most commonly monitored variable is left ventricular ejection fraction (LVEF). Preliminary data suggests that the use of left ventricular end-systolic volume (LVESVi) and left ventricular end-diastolic volume (LVEDVi) may be more appropriate for describing changes in LV remodeling (45). In addition, reversal of changes in cardiac remodeling after initiation of sacubitril/valsartan were associated with a reduction in NT-proBNP after initiation of therapy. Taken together, this reduction along with an improvement in echocardiography is a key indicator of reverse LV remodeling and associated with improved clinical outcomes. Reverse remodeling has become a therapeutic target in HF. Therefore, this article aims to analyze the extent to which sacubitril/valsartan improves biomarkers and echocardiographic parameters of reverse cardiac remodeling in patients with HF after AMI.

Angiotensin-converting enzyme inhibitors/angiotensin II receptor antagonists (ACEI/ARBs), beta-blockers, and aldosterone receptor antagonists (MRAs) are the mainstays of treatment for patients with LVSD after myocardial infarction (46). Whether sacubitril and valsartan can be used early in patients with heart failure after acute myocardial infarction has not yet been fully confirmed. This systematic review and meta-analysis of 13 studies, including 10 RCTs and 3 non-RCTs in 6968 patients with new-onset acute MI and HF. We demonstrate that sacubitril-valsartan not only delays Ventricular remodeling, reducing the level of heart failure biomarkers but also can further reduce the overall incidence of adverse cardiovascular events, especially the heart failure readmission rate, in patients with AMI and HF.

In terms of echocardiographic results, the meta-analysis results showed that the experimental group could effectively increase the LVEF within 6 months of follow-up. Compared with ACEI/ARB group, LVESVI and LVEDVI in the sacubitril-valsartan group were significantly reduced. The results of reducing LVEDD in the experimental group were significantly higher than those in the control group at 3, 6, and 12 months of follow-up. Therefore, sacubitril and valsartan can actively and effectively prevent and control ventricular remodeling and delay the occurrence and development of heart failure.

In terms of clinical outcomes, the meta-analysis results showed that sacubitril/valsartan effectively increased the 6-min walk test distance. Regarding major adverse cardiac events (MACEs), sacubitril/valsartan has a lower hospitalization rate for recurrent HF and a lower overall adverse event rate than ACEI/ARB, but no significant difference in cardiac death, recurrent MI, and malignant arrhythmia compared with ACEI/ARB. This may be due to an insufficient number of included studies and inconsistent follow-up time. Therefore, more large-scale and high-quality RCTs are needed to more clearly observe specific results. In conclusion, the addition of sacubitril and valsartan sodium on the basis of conventional treatment in patients with MI complicated with mental failure can further improve clinical efficacy.

In terms of biomarkers, sacubitril-valsartan decreased NT-proBNP levels compared with the control group. Furthermore, there was no significant difference in the 12-month follow-up, but the effect was better in the 6 and 3-month follow-ups. This may be because not enough included literature mentioned 12 month follow-ups, and had no major guiding significance for clinical practice. Due to the limited amount of included literature and data, in order to achieve new breakthroughs in future trials, we did not comparatively analyze the inflammatory factors and markers of MI. In addition, regarding safety, the incidence of ARNI cough in the experimental group was lower than in the control group. However, the incidence of hypotension was higher, and it did not increase the side effects of hyperkalemia, angioedema, and renal insufficiency. In general, sacubitril and valsartan have high safety and no obvious adverse reactions. However, it should be noted that blood pressure monitoring should be a focus during clinical use.

### Limitation

Although the studies included in this article were of reasonably high quality, our study had a number of drawbacks. First, the type of heart failure included in the research was not explicitly defined, which decreased the accuracy of the findings. Second, the follow-up duration of the included trials ranged from 1 to 12 months, allowing for the evaluation of short- and medium-term follow-up outcomes but not long-term follow-up results. Third, not all patients took the study drug at the prescribed dose, and we did not examine combined drug concentrations. However, based on the analysis of the PIONEER-HF study, sacubitril and valsartan appear to be safe and beneficial regardless of the dose level attained in the first month following hospitalization (47). Fourth, three groups in the control group were relatively blank control groups, making it difficult to compare ARNI with ACEI/ARB in some measures.

## Conclusion

Current evidence shows that sacubitril-valsartan can reduce heart failure biomarkers, reverse and delay ventricular remodeling, improve patients’ quality of life, and improve the long-term prognosis of MI during short- and medium-term follow-up. Therefore, the use of ARNI in treating patients with AMI and HF has significant clinical prospects.

## Data availability statement

The original contributions presented in this study are included in the article/Supplementary material, further inquiries can be directed to the corresponding author.

## Author contributions

XZ and HZ: conception and design. YZ: provision of study materials or patients. XZ, XYT, and XDT: collection and assembly of data. XZ: data analysis and interpretation. All authors: manuscript writing and final approval of manuscript.
